# What Factors Lead to Racial Disparities in Outcomes After Total Knee Arthroplasty?

**DOI:** 10.1007/s40615-021-01168-4

**Published:** 2021-10-12

**Authors:** Daniel A. Hu, James B. Hu, Ariel Lee, William J. Rubenstein, Kevin M. Hwang, Said A. Ibrahim, Alfred C. Kuo

**Affiliations:** 1 Northwestern University Feinberg School of Medicine, Chicago, IL USA; 2 Irvine School of Medicine, University of California, Irvine, CA USA; 3 Department of Orthopaedic Surgery, University of California, San Francisco, CA USA; 4 Department of Healthcare Policy and Research, Weill Cornell Medicine, New York City, NY USA; 5 Orthopedic Surgery Section, San Francisco Veterans Affairs Health Care System, San Francisco, CA USA

**Keywords:** Total knee arthroplasty, Clinical outcomes, Racial health disparity, Readmission

## Abstract

Total knee arthroplasty (TKA) is one of the most commonly performed, major elective surgeries in the USA. African American TKA patients on average experience worse clinical outcomes than whites, including lower improvements in patient-reported outcomes and higher rates of complications, hospital readmissions, and reoperations. The mechanisms leading to these racial health disparities are unclear, but likely involve patient, provider, healthcare system, and societal factors. Lower physical and mental health at baseline, lower social support, provider bias, lower rates of health insurance coverage, higher utilization of lower quality hospitals, and systemic racism may contribute to the inferior outcomes that African Americans experience. Limited evidence suggests that improving the quality of surgical care can offset these factors and lead to a reduction in outcome disparities.

## Introduction

Total knee arthroplasty (TKA) is one of the most common major surgeries performed in the USA and accounts for a large proportion of Medicare surgical expenditures. Although most patients experience improved symptoms and function after surgery, patients from racial and ethnic minorities often experience worse outcomes after surgery than white patients. Compared to whites, African American TKA patients have been reported to experience higher rates of complications, readmissions, revision surgery, and mortality as well as smaller improvements in patient-reported outcome measures (PROMs) [1–5]. These results are found in a wide range of health care systems across the USA. Black TKA patients of Kaiser Permanente, one of the largest private, nonprofit healthcare plans in the USA, have higher rates of emergency department visits, readmissions, and aseptic revision surgery than white patients [6]. In a study of TKAs performed in the State of Pennsylvania, Blum et al. also demonstrated that black patients had higher rates of revision surgery [7]. Outcome disparities exist for patients of the Veterans Health Administration (VHA), even though the VHA is an equal-access system that provides care to veterans regardless of race, ethnicity, income, or other socioeconomic factors. Ibrahim et al. found that black VHA TKA patients experienced higher complication rates than whites [8]. More recently, we have found that black VHA TKA patients have lower improvements in PROMs and higher rates of revision surgery [9, 10].

There is no consensus as to why these disparities exist, and they are unlikely to arise from a single cause. The goal of this review is to describe the interrelated patient, provider, healthcare system, and societal factors that likely contribute to the poorer outcomes of black patients compared to whites. An understanding of the causes of disparities is required to guide actions to reduce unequal outcomes.

## Patient Factors

Multiple patient-specific factors may contribute to racial disparities in clinical outcomes following TKA. The poorer health of the black population compared to whites is likely to be a key contributor. According to 2015 data from the Behavioral Risk Factor Surveillance System, African Americans have higher rates of medical conditions such as obesity, hypertension, diabetes mellitus, and history of stroke than whites across all age groups [11]. Medical comorbidities are associated with poorer surgical outcomes and increased complication rates following TKA. For example, diabetes mellitus and super obesity are independent risk factors for surgical site infection after upper and lower extremity arthroplasty [12]. In a study of the National Inpatient Sample, Hustedt et al. demonstrated that patients with more comorbidities had higher rates of postoperative complications following total hip and knee arthroplasties [13]. Congestive heart failure, valvular heart disease, and chronic obstructive pulmonary disease were associated with the highest risks of major complications. Elmallah et al. found in a cohort of 283 TKA patients that those with hypertension, cardiovascular disease, endocrine disease, and gastrointestinal disease had worse postoperative PROM results compared to those without these comorbidities [14].

Differences in preoperative psychologic health may also contribute to racial disparities after TKA. Adverse baseline psychological health increases the likelihood of poor outcomes following TKA. In a systematic review of 19 publications, including ten from the USA, psychological health was found to be a significant predictor of pain, function, or satisfaction after TKA in 16 studies [15]. Patients with preoperative anxiety or severe depression experience greater pain 1 year after TKA than patients without these conditions [16]. These effects can persist over time and preoperative depression is associated with lower knee PROM scores 5 years after surgery [17]. Although African Americans have a lower or equivalent incidence of mental illness compared to the general population, blacks often experience more severe psychiatric illness [18] and commonly experience higher levels of psychological distress [19]. In addition, black Americans with mood and anxiety disorders have a higher risk of experiencing persistent symptoms compared to whites [20]. Finally, African Americans are less likely to utilize mental health services than whites [21], which may be due to lack of health insurance, internalized stigma to seeking care for mental health issues, and/or distrust of medical providers.

Perceived poor social support can also affect mental health outcomes [22] and differences in social support may also contribute to racial disparities above and beyond their direct effects on mental health. Social isolation is associated with negative physical and mental health outcomes across races [23]. In a survey of 799 patients with knee osteoarthritis (OA), African Americans were less likely to be married, reported fewer close friends, and had lower Medical Outcomes Study-Social Support Scale (MOS-SSS) scores compared to whites [24]. Similarly, in a survey of 909 male VHA patients with moderate or severe hip or knee OA, the mean MOS-SSS score for black patients was significantly lower than for white patients [25]. The MOSS-SSS consists of four functional support scales (tangible, emotional/informational, affectionate support, and positive social interaction) [26]. Less tangible support has in turn been associated with worse Western Ontario McMaster function scores after TKA [27].

The socioeconomic status (SES) of the black population is another contributor to racial differences in outcomes [28]. Data from the 2019 Survey of Consumer Finances shows marked, persistent racial disparities in family wealth in the USA, with white families having eight times the wealth of black families [29]. For Americans less than 65 years of age, blacks have higher rates of unmet health care needs secondary to cost, lower rates of health insurance coverage, higher rates of public health insurance, and lower rates private health insurance compared to whites [30, 31]. Race, SES, and insurance status interact to affect outcomes after surgery. Multivariate analysis of state administrative data shows that black race, higher comorbidity burden, lower income, and public payer status (Medicare and Medicaid) are all associated with significantly higher rates of readmission after TKA [32]. Racial disparities in health insurance coverage will be further examined in the discussion on healthcare system effects.

## Provider and Healthcare System Factors

Provider bias is hypothesized to be another contributor to racial disparities after TKA. Bias is defined as a prejudice for or against a person, thing, or idea. Biases range from overt prejudices and stereotypes to subtle attitudes that can affect perception, behaviors, and decision-making. Implicit biases are unconscious attitudes and associations that are automatically triggered when thinking about specific social groups [33]. Although most providers strive to avoid conscious biases while treating patients, it is harder to avoid unconscious, implicit biases. The implicit association test (IAT), a validated tool measuring the strength of association between concepts, has been widely used to measure implicit bias among providers [34]. The IAT pairs different photos of black and white people with “pleasant” or “unpleasant” words. The faster and more accurately the participant responds to the pairings, the stronger the association. For instance, a person with an implicit pro-white bias will pair a photo of a smiling white person with “pleasant” words such as beautiful and happy more quickly and accurately than a photo of a smiling black person. Multiple studies have found evidence of implicit racial bias among healthcare providers as well as an association between implicit racial bias and inferior communication between patients and providers [34, 35]. Although the literature is clear that racial implicit bias impacts patient-provider interactions, there is less clarity regarding the impact on clinical outcomes [36]. There is little data regarding bias and a surgeon’s decision to offer treatment in real-world settings. For example, racial disparities in outcomes may arise in part if surgeons are more willing to offer TKA to high-risk African American patients with multiple comorbidities than to similar white patients. Future studies must examine the relationships between provider bias and patient outcomes in real-life scenarios rather than in standardized patient cases or theoretical vignettes.

Provider and healthcare system quality are also likely key contributors to racial disparities in TKA outcomes. Disparities in healthcare outcomes between minority populations and whites are generally not due to worse quality of care within the same hospital. In a study examining inpatient discharge data across thirteen states and comparing risk-adjusted quality indicators by race and ethnicity, only a small subset of hospitals was found to provide systematically lower quality care to minority groups [37]. Instead, African American patients tend to undergo surgery at lower-quality hospitals than whites. Analysis of Medicare data from 2005 to 2008 demonstrated that while African American patients lived closer to higher-quality hospitals (assessed with composite measures factoring in risk-adjusted morbidity and outcomes) than white patients, they were more likely than whites to undergo major surgery (coronary artery bypass, abdominal aortic aneurysm repair, and lung cancer resection) at lower-quality hospitals [38]. Use of lower-quality hospitals was strongly associated with higher degrees of racial residential segregation. Similarly, analysis of records of elderly Medicare beneficiaries who received TKAs from 2002 to 2005 showed that African American patients were more likely than their white counterparts to be admitted to hospitals with higher risk-adjusted rates of postoperative complications or mortality [39]. Data on hospital TKA volume also suggests that black patients often undergo surgery in lower-quality institutions. Total hip and knee arthroplasty volume correlates with composite hospital quality scores [40] and African Americans are less likely to undergo TKA in high-volume hospitals than white Americans [4].

Disparities in health insurance coverage may also contribute to the higher utilization of lower-quality hospitals by African American patients. Uninsured and underinsured patients often receive care at safety-net hospitals [41], which frequently demonstrate poor performance on quality measures such as readmissions [42]. In 2014, 20.7% of black nonelderly adults were uninsured compared to 11.7% of whites [31]. In addition, 26.4% of black patients and 13.9% of white patients had public health insurance. Medicaid payer status has been found to be an important factor in predicting total joint arthroplasty outcomes. For example, in a systematic review by Lakomkin et al., seven analyses identified a significant association between Medicaid coverage and short-term readmissions, and two analyses demonstrated a relationship between prolonged length of stay and Medicaid insurance status [43].

## Societal Factors

Multiple governmental and medical organizations in the USA, including the National Institutes of Health, have concluded that structural racism contributes to racial health disparities [44]. Systemic racism is defined as policies and practices in institutions, including those in health care, housing, employment, and education, that lead to the exclusion or promotion of specific groups [45, 46]. It can be divided into two categories, institutional and structural racism. Institutional racism specifically occurs within and between institutions and consists of discriminatory treatment and differences in opportunities based on race, perpetuated by institutions such as schools and hospitals. One example is the legalized racial segregation of medical and other facilities by Jim Crow laws. Structural racism occurs within a society and encompasses the history of a particular nation, such as culture which normalizes and legitimizes racism, and the institutions (housing, education, employment, earnings, and health care) which reinforce and perpetuate racism. Importantly, structural racism does not comprise the actions or intent of specific individuals, but rather exists on a macro-scale [47].

There are as of yet few papers on the effects of systemic racism in orthopedics, much less the effects of systemic racism on TKA outcomes. Structural racism can affect health through multiple pathways, including racial residential segregation. Discriminatory practices present today in more subtle ways than in previous decades, and overt methods by which racial segregation was historically achieved, such Jim Crow laws, are rare or absent. Current methods are less conspicuous, such as the use of “racial steering” for segregating neighborhoods [47]. In this practice, prospective home buyers are guided by real estate brokers toward or away from certain neighborhoods based on the buyers’ race. Racial residential segregation has been hypothesized to influence health outcomes by affecting health system infrastructure, including availability of health care services and facilities, and by affecting neighborhood context, including the social, physical, economic, and environmental factors that influence well-being [48, 49]. The concentration of minorities into less desirable and lower SES neighborhoods correlates to exposure to pollutants, decreased longevity, increased risk of chronic disease, and higher crime rates [46, 47]. Societal conditions therefore contribute to the patient, provider, and healthcare system factors that drive disparities in outcomes.

## Conclusion

African American patients are more likely to experience worse outcomes after TKA than white patients. The reasons for this are multifactorial and complex, and likely include disparities in physical and mental health, unequal access to quality healthcare, and structural inequities in society. Just as there is no single, simple reason for these unequal outcomes, there is no single, simple solution. The findings of Aseltine et al. offer some hope [50]. Using claims data from the State of Connecticut, they estimated the rates of 30-day admissions after total hip and knee arthroplasty from 2005 to 2015. As with previous studies, they found that African Americans had higher readmission rates than whites at the beginning of the study period. By 2015, readmission rates had declined for all races, and the difference between black and white patients had narrowed. Although the authors were unable to identify the reasons for this reduction in readmission disparity, there is evidence suggesting that improving the quality of surgical care may reduce health care disparities. In a single-institution study of postoperative length of stay after colorectal surgery, racial disparities in lengths of stay were eliminated after implementation of an enhanced recovery after surgery pathway [51]. Further research is required to fully understand the processes that lead to disparities after TKA and to design programs to reduce these disparities.

## Data Availability

Not applicable.
